# Ambulatory Antibiotic Use Patterns in Bolivia: Identifying Targets for Future Antibiotic Stewardship Efforts in Latin America

**DOI:** 10.1017/ash.2023.411

**Published:** 2023-08-14

**Authors:** Rodolfo E. Quirós, Maria E. Mesalles, Elvio D. Escobar, Juan Carlos Tapia Torrez, Sara E. Cosgrove, Valeria Fabre

**Affiliations:** 1 Sanatorio Las Lomas, Buenos Aires, Argentina; 2 Department of Medicine, MedStar Hospital, Baltimore, MD, USA; 3 Clínica Ángel Foianini, Santa Cruz de la Sierra, Bolivia; 4 Department of Medicine, Division of Infectious Diseases, Johns Hopkins University School of Medicine, Baltimore, MD, USA

## Abstract

We evaluated antibiotic use in a private health insurance network in Bolivia with two different healthcare plans. The Health Maintenance Organization plan had 29% lower antibiotic consumption and fewer broad-spectrum antibiotics prescribed than the Preferred Provider Organization. Furthermore, we identified potential targets for future antibiotic stewardship efforts.

It is estimated that 60%–90% of human antibiotic use occurs in the outpatient setting.^1^ In the United States, approximately 50% of ambulatory prescriptions are inappropriate.^2^ Similar findings were observed in resource-limited settings, where >50% of antibiotic prescriptions in primary care were inappropriate, mostly due to treatment of viral illnesses.^3^ Understanding patterns and drivers of antibiotic use is important to inform effective antibiotic stewardship (AS) interventions. In high-income countries, several factors have been associated with higher antibiotic use including geographic region, provider specialty, health plan, and patient income.^4,5^ These data are lacking for Latin America, where most studies have focused on inpatient antibiotic use.^6^

We sought to investigate and describe ambulatory antibiotic use in a private health network in Bolivia with two different health plans, a Health Maintenance Organization (HMO) and a Preferred Provider Organization (PPO) plans.

## Methods

We conducted a retrospective observational study of ambulatory antibiotic use among all patients of a private healthcare insurance network in Santa Cruz de la Sierra, Bolivia (the country’s most populous city) between January 2017 and December 2018. The network includes two business plans, an HMO plan where care is coordinated by a general practitioner (GP) and a PPO plan in which patients can access specialists directly without a referral from a GP. While the PPO plan was slightly more expensive than the HMO plan; antibiotic co-pays were similar for both plans (usually a 50% medicine co-pay for both plans). Patients without a prescription are responsible for 100% of the medication cost. There were no incentives for physicians in either business plan to promote less or more antibiotic prescriptions during the study period.

During the 24-month study period, there were 8,405 patients in the network, including 2,419 patients in the HMO plan and 5,986 patients in the PPO plan.

Data were extracted from pharmacy records (dispensed antibiotics), and antibiotic use was calculated as daily defined doses (DDD) per 1,000 patient-days. Antibiotics from the J01 group from the Anatomical Therapeutic Chemical (ATC)-World Health Organization (WHO) classification system which includes antibacterials used systemically (excluding anti-mycobacterials)^7^ were included in the study. Antibiotics were further grouped using WHO’s *AWaRe* (*Access*, *Watch,* and *Reserve*) antibiotic classification.^8^ *Access* includes antibiotics such as amoxicillin, amoxicillin-clavulanic acid, first-generation cephalosporins, doxycycline, and trimethoprim-sulfamethoxazole. *Watch* antibiotics includes macrolides, second-, third-, and fourth-generation cephalosporins, fluoroquinolones, and aminoglycosides. We used descriptive statistics and the Byar test with 95% confidence intervals (CIs) for differences in DDD between different age, plan, and gender groups. A 2-sided *P* value < .05 was considered statistically significant for all tests. Statistical analysis was performed using Openepi.^8^

The study was deemed exempt by the local Institutional Review Board.

## Results

The mean age of HMO and PPO patients was 23.8±standard deviation (SD) 15.5 and 26.3 ± SD 16.9, respectively (*P* < .01). Females represented 56.1% (1,357/2,419) and 52.9% (3,172/5,986) of HMO and PPO patients, respectively (*P* = .01). Thirty-six percent (881/2,419) and 33.6% (2,010/5,986) of HMO and PPO patients, respectively, were children (*P* = .08).

**Figure 1.**
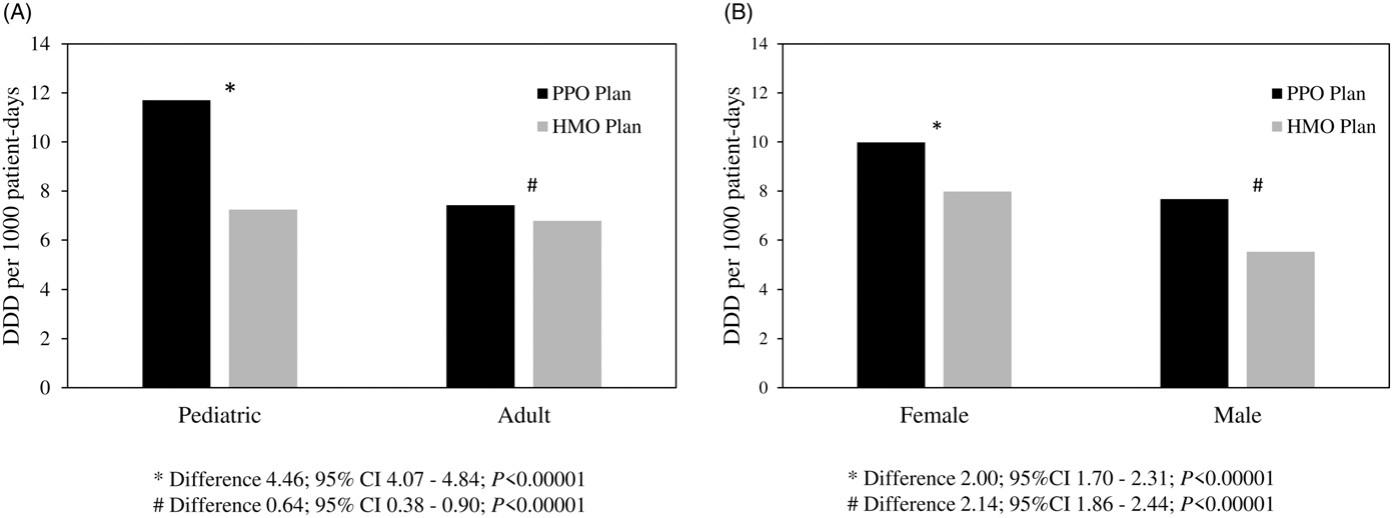
Ambulatory antibiotic consumption in daily defined doses (DDD) per 1,000 patient days by the Preferred Provider Organization (PPO) and the Health Maintenance Organization (HMO) plans for adult and pediatric patients (A) and for female and male patients (B).

Antibiotic consumption was higher in the PPO plan compared to the HMO plan (8.89 vs 6.95 DDD/1,000 patient-days [PD], difference 1.94, 95% CI: 1.72–2.15, *P* < .001), for both female (9.98 vs 7.98 DDD/1,000 PD in PPO and HMO, respectively, difference 2.0 95% CI: 1.7–2.31, *P* < .001) and male patients (7.67 vs 5.52 DDD/1,000 PD in PPO and HMO, respectively, difference 2.15, 95% CI: 1.72–2.15, *P* < .001), and for pediatric patients (11.7 vs 7.24 DDD/1,000 PD in PPO and HMO, respectively, difference 4.46, 95% CI: 4.07–4.84, *P* < .0001), Figure 1.

The two most prescribed antibiotics for both plans were macrolides and amoxicillin-clavulanic acid. Overall, 55% of antibiotics were in the *Access* group. The PPO plan had higher consumption of *Watch* antibiotics (including fluoroquinolones, macrolides, and third-generation cephalosporins) compared to the HMO plan (46.9% vs 37.6%, respectively, *P* < .001), Table 1. There was a higher proportion of *Watch* antibiotics in children versus adults (48.2% vs 41.9%, respectively, *P* < .001), which was mostly driven by macrolides. GPs prescribed 27% of HMO and 7% of PPO antibiotics. Pulmonary, gastrointestinal, and ears/nose/throat physicians prescribed 3%, 5%, and 13% of PPO vs 0%, 2%, and 7% of HMO antibiotics.

**Table 1.** Ambulatory Antibiotic Consumption in HMO versus PPO Plans of a Closed Private Health Network in Bolivia

Antibiotic group*	DDD per 1,000 member-days			95% CI	*P* value
	HMO	PPO	Diff.		
Total	6.95	8.89	−1.94	−2.15 to −1.72	.0000
Penicillins+β-lactamase inhibitors† (*A*)	2.65	2.85	−0.20	−0.32 to −0.07	.0032
Macrolides (*W*)	1.17	2.46	−1.29	−1.38 to −1.19	.0000
Penicillins with extended spectrum‡ (*A*)	0.79	0.90	−0.11	−0.19 to −0.05	.0018
Fluoroquinolones (*W*)	0.72	0.84	−0.12	−0.19 to −0.05	.0007
3^rd^ gen Cephalosporins (*W*)	0.21	0.65	−0.44	−0.49 to −0.39	.0000
Tetracyclines (*A*)	0.63	0.38	0.25	0.20 to 0.31	.0000
Sulfamethoxazole + trimethoprim (*A*)	0.40	0.37	0.03	−0.01 to 0.08	.1732
Nitrofurantoin (*A*)	0.15	0.14	0.01	0.03 to −0.03	.7804
1^st^ gen Cephalosporins (*A*)	0.07	0.17	−0.10	−0.13 to −0.08	.0000
Aminoglycosides (*A*)	0.07	0.04	0.03	0.02 to 0.06	.0000
β-lactamase-resistant penicillins^¥^ (*A*)	0.02	0.05	−0.03	−0.04 to −0.02	.0000
β-lactamase-sensitive penicillins^€^ (*A*)	0.05	0.02	0.03	0.01 to 0.04	.0002
Lincosamides (clindamycin) (*A*)	0.02	0.02	0.00	0.01 to −0.01	.5873

(*W*), denotes *Watch*, (A), denotes *Access* category of *AWaRe* classification; HMO, Health Maintenance Organization; PPO, Preferred Provider Organization, DDD, defined daily doses.

* Using J01 group ATC-WHO classification.

† Includes amoxicillin-clavulanic acid.

‡ Includes ampicillin, amoxicillin.

¥ Includes dicloxacillin, cloxacillin.

€ Includes phenoxymethylpenicillin, benzathine benzylpenicillin, procaine benzylpenicillin.

## Discussion

Evaluation of antibiotic use within a private healthcare network in Bolivia revealed disparities between health plans, with higher antibiotic consumption and more broad-spectrum antibiotic use in the PPO plan compared to a GP-coordinated HMO plan. Similar findings were reported in a study of antibiotic use in that included 360 health plans in the United States where HMO plans had lower prescription rates of antibiotics for acute bronchitis in adults, and more appropriate antibiotic use in children presenting with upper respiratory tract infections compared to PPO plans.^4^ We speculate having care coordinated by a GP in the HMO plan may contribute to lower antibiotic use by HMO plan patients as GPs are more likely to provide longitudinal care increasing the chances of making more appropriate antibiotic decisions than specialists who are more likely to have occasional interactions with patients. While treatment guidelines which include recommendations for outpatients with common infectious syndromes were available to all physicians within the network, it remains unclear if lack of adherence was a major driver of the differences observed between the two plans. Female patients are more likely to receive antibiotics in their lifetime than male patients^9^; however, sex alone would not explain antibiotic consumption differences as antibiotic consumption was also higher among PPO male patients.

WHO established the *AWaRe* classification of antibiotics based on their impact on AMR as an AS tool.^8^ While all antibiotics increase the risk of colonization or infection with multidrug-resistant bacteria, patients exposed to *Watch* antibiotics have a higher risk of acquiring MDR bacteria than those exposed to *Access* antibiotics.^9^ WHO recommends *Access* antibiotics to represent at least 60% of all prescriptions at the country level. Bolivia has adopted the *AWaRe* classification in their essential national medication list and prohibits medication sales without a prescription; however, a national AMR action plan has not yet been implemented, and education campaigns about the risks of inappropriate antimicrobial use have been limited, according to recent WHO Tripartite AMR Country Self-Assessment Survey results. In our cohort, *Access* antibiotics did not reach the minimum target, with a larger gap for the PPO plan. We observed a higher proportion of *Watch* antibiotics among children compared to adults. This is in agreement with previous studies in resource-limited settings where increased use of *Watch* antibiotics was associated with patient characteristics (e.g., younger age and pulmonary comorbidities) and non-patient factors such as pharmacy-drug availability.^10^ The later probably did not play a major role in our cohort since our patients would have had similar pharmacy access; however, patient demands for antibiotics could have differed between plan patients.

Limitations to this study include the lack of patient- and visit-level data including patient comorbidities and visit diagnoses which are not electronically available. Although our study did not explore a comprehensive list of drives of antibiotic prescribing, it highlights the need to strengthen AS efforts in ambulatory care, especially in the pediatric population, and to improve engagement of specialists in AS. Additional studies to better understand “antibiotic behavior” by physicians and patients in the different plans would be beneficial to refine AS initiatives at improving antibiotic use in the region.
